# SemaTyP: a knowledge graph based literature mining method for drug discovery

**DOI:** 10.1186/s12859-018-2167-5

**Published:** 2018-05-30

**Authors:** Shengtian Sang, Zhihao Yang, Lei Wang, Xiaoxia Liu, Hongfei Lin, Jian Wang

**Affiliations:** 1 College of Computer Science and Technology, Dalian University of Technology, Hongling Road, Dalian, 116023 China; 2 Beijing Institute of Health Administration and Medical Information, Beijing, 100850 China

**Keywords:** Literature-based discovery, Knowledge graph, Drug discovery, Literature mining

## Abstract

**Background:**

Drug discovery is the process through which potential new medicines are identified. High-throughput screening and computer-aided drug discovery/design are the two main drug discovery methods for now, which have successfully discovered a series of drugs. However, development of new drugs is still an extremely time-consuming and expensive process. Biomedical literature contains important clues for the identification of potential treatments. It could support experts in biomedicine on their way towards new discoveries.

**Methods:**

Here, we propose a biomedical knowledge graph-based drug discovery method called SemaTyP, which discovers candidate drugs for diseases by mining published biomedical literature. We first construct a biomedical knowledge graph with the relations extracted from biomedical abstracts, then a logistic regression model is trained by learning the semantic types of paths of known drug therapies’ existing in the biomedical knowledge graph, finally the learned model is used to discover drug therapies for new diseases.

**Results:**

The experimental results show that our method could not only effectively discover new drug therapies for new diseases, but also could provide the potential mechanism of action of the candidate drugs.

**Conclusions:**

In this paper we propose a novel knowledge graph based literature mining method for drug discovery. It could be a supplementary method for current drug discovery methods.

**Electronic supplementary material:**

The online version of this article (10.1186/s12859-018-2167-5) contains supplementary material, which is available to authorized users.

## Background

Drug discovery is the process through which potential new medicines are identified. High-throughput screening (HTS) and computer-aided drug discovery/design (CADD) are the two main drug discovery methods for now [1]. Despite advances in technology and understanding of biological systems, drug discovery is still a lengthy and expensive process with low rate of new therapeutic discovery [2, 3]. Developing a new drug is estimated to take 14 years and cost approximately $1.8 billion [4]. In contrast, Literature-Based Discovery (LBD) is a safe and low-cost approach to identify new drugs for indications. LBD seeks to discover new relationships in existing knowledge from unrelated literatures [5]. Drugs are often discovered on the serendipitous observation that a drug effect may be therapeutically useful if it induces a desired effect or counters a disease phenotype [6]. For instance, Don R. Swanson (1924–2012) proposed fish oil as a new treatment for Raynaud’s disease in 1986 after noting the association “high blood viscosity is observed among Raynaud’s Syndrome sufferers” in some biomedical articles and another association “dietary fish oil lowers blood viscosity” in other articles [7]. This hypothesis was verified in medical experiments two years later. Basic LBD techniques search for a set of intermediate terms that frequently co-occur with a source term and a target term [5]. As shown in the above example, “blood viscosity” is the intermediate term in associating the “dietary fish oil” with the “Raynaud’s Syndrome”. In addition, more sophisticated LBD methods first employ natural language processing (NLP) techniques to extract relations between entities from biomedical literature. Then novel discoveries could be analyzed from the extracted relations [8]. For example, Hristovski et al. used SemRep to extract relations among entities from biomedical literature [9]. These extracted relations could then be used for inferring novel relationships in literatures [8]. More recently, a number of recent LBD methods have explored methods that utilize certain graph data structures. For example, Cameron et al. introduced a graph-based method that automatically finds clusters of contextually similar paths in a semantic graph [10, 11]. These clusters are used to elucidate the latent associations between disjoint concepts in the literatures. These existing LBD methods have several limitations. The main issue of of term co-occurrence approach is that the extracted relationships lack logical explanations[12]. NLP-based methods strongly depends on the availability of domain-specific NLP tools [13]. Graph-based methods don’t consider the different semantic types of nodes in the graph. Most importantly, all existing methods have not exploited all available published biomedical literature for drug discovery. They only focus on part of the abstracts related to disease of interest. This could lead to missing the valuable informations existing in the filtered literature.

In this paper, we propose a biomedical knowledge graph based inference method to discover drug therapies from literature. Knowledge graphs (KGs) are collections of relational facts, which have proven to be sources of valuable information that have become important for various applications [14]. The famous knowledge graphs include Freebase [15], DBpedia [16], Nell [17] and YAGO [18], etc. Here, we first construct a biomedical knowledge graph called SemKG with relations extracted from PubMed abstracts. Then based on SemKG, a drug discovery method called SemaTyP (Semantic Type Path) is introduced to exploit the semantic types of paths to discover drug therapies. The experimental results show that our method could not only discover new candidate drugs for new diseases, but also could provide the mechanism of action of the candidate drugs. To summarize, the contributions of the paper is: First, we introduced a biomedical knowledge graph - SemKG - which is constructed by integrating information extracted from PubMed abstracts. Second, this is the first method that discovers candidate drugs by using biomedical knowledge graph. Our method could be a supplementary method for current drug discovery methods, which could improve the successfulness in discovering new medicine for recently incurable diseases.

## Methods

### Materials and tools

The biomedical knowledge graph used in this study is constructed based on the predications (subject-relation-object triples) extracted from PubMed abstracts by SemRep. In this section, the datasets and tools used in this study are briefly introduced.

#### PubMed

PubMed is a free search engine accessing primarily the MEDLINE database of references and abstracts on life sciences and biomedical topics. It provides now access to more than 26 million citations, adding thousands of records daily [19].

#### UMLS semantic network

The Unified Medical Language System (UMLS) semantic network consists of 133 semantic types and 54 relationships that exist between the semantic types. In this paper, the abbreviations are adopted to represent the semantic types. For example, ‘podg’ represents ‘Patient or Disabled Group’ and ‘topp’ is ’Therapeutic or Preventive Procedure’.

#### Metamap

MetaMap is a widely available program providing access from biomedical text to the concepts in the unified medical language system (UMLS) Metathesaurus [20]. It could be applied for biomedical name entity recognition, word sense disambiguation (WSD) and other natural language processing tasks [21].

#### SemRep

SemRep is a relation extraction tool which first uses MetaMap to map noun phrases to UMLS concepts [22] then extracts semantic predications from biomedical free text [23]. For example, from the sentence “We used hemofiltration to treat a patient with digoxin overdose that was complicated by refractory hyperkalemia”, SemRep extracts four predications:

1. Hemofiltration |**topp** TREATS Patients |**podg**
2. Digoxin overdose |**inpo** PROCESS_OF Patients |**podg**
3. Hyperkalemia |**patf** COMPLICATES Digoxin overdose |**inpo**
4. Hemofiltration |**topp** TREATS(INFER) Digoxin overdose |**inpo**

On the right of symbol ‘ |’ is the abbreviation of entity’s semantic type (black bold).

### Construction of SemKG

**Fig. 1:**
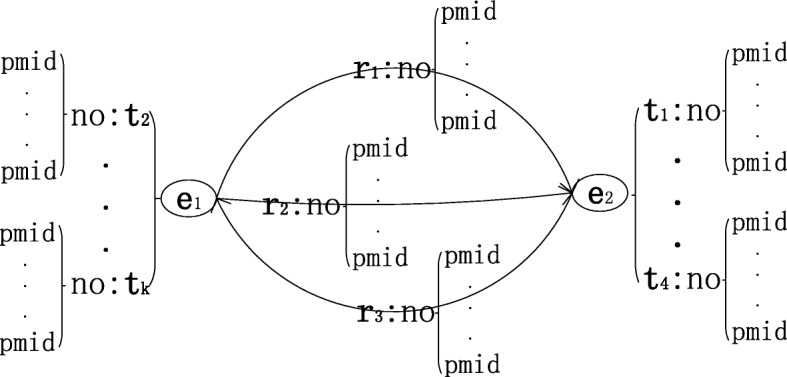
The prototype example of SemKG. The symbol e, r and t represent entity, relation and the type of the entity, respectively. no is the number of occurrences and pmid is PubMed ID

**Fig. 2:**
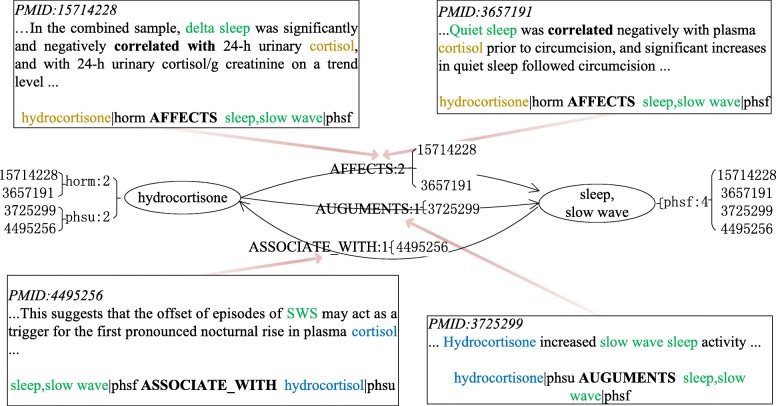
An illustration of one edge in SemKG

Knowledge graph is a multi-relational graph composed of entities as nodes and relations as different types of edges. In this work, we constructed a biomedical knowledge graph, called SemKG, with the predications which are extracted from PubMed abstracts by SemRep. In the SemKG, let *E*={*e*_1_,*e*_2_,…,*e*_*N*_} denote the set of n entities, *R*={*r*_1_,*r*_2_,…,*r*_*M*_} denote the set of relations between entities and *T*={*t*_1_,*t*_2_,…,*t*_*K*_} denote semantic type of entities. The elements of R and T are all from the UMLS semantic network. The edge between entities *e*_*i*_ and *e*_*j*_ is weighted by the number of predications that have been extracted. Besides, the attribute of edge includes the abstracts’ PubMed ID (pmid) from where the predications are extracted. A prototype example of the SemKG is illustrated in Fig. 1. Figure 2 is an illustration of an edge of the SemKG, it shows that there are three different relations between “hydrocortisone” and “sleep, slow wave” which are extracted from four abstracts (pmid 15714228, 3657191, 3725299 and 4495256). The relation “AFFECTS” is extracted from two abstracts (pmid 15714228 and 3657191) simultaneously. Figure 2 shows the same entity could be assigned with different semantic types. For example, the “hydrocortisone” is a kind of “hormone” (horm) in the predications extracted from the two abstracts (pmid 15714228 and 3657191) and it also could be “Pharmacologic Substance” (phsu) in other predications (pmid 4495256).

### SemaTyP method

#### Path exploration

Given a knowledge graph KG, a path *π* is defined as a sequence of predications $\phantom {\dot {i}\!}e_{0}r_{0}e_{1}r_{1}\ldots {r}_{\ell -1}e_{\ell }$, where *ℓ* is the length of path *π*. For a gold standard *drug*_*i*_−*target*_*i*_−*disease*_*i*_ case, which provides information about targeted disease _*i*_ and the corresponding drug _*i*_ directed at the target _*i*_. SemaTyP first constructs training data by obtaining all paths $\pi ^{\ell }=\rho (drug_{i}\rightarrow disease_{i};target_{i},\ell)\phantom {\dot {i}\!}$, which encodes a path of length *ℓ* reaching node disease _*i*_ from source node drug _*i*_ and crossing node target _*i*_. Then $\mathbf {p}_\ell =\left \{\pi ^{\ell }_{1},\pi ^{\ell }_{2},\pi ^{\ell }_{3},\pi ^{\ell }_{4}\ldots \right \}\phantom {\dot {i}\!}$ is the set of all *ℓ* length paths. All paths in $\P =\{\mathbf {p}_{2},\mathbf {p}_{4},\mathbf {p}_{5},\ldots,\mathbf {p}_\ell \}\phantom {\dot {i}\!}$ are considered as positive training data. The minimum length of path in *¶* is 2, which represents the path *drug*_*i*_−*target*_*i*_−*disease*_*i*_. Similarly, the corresponding negative training data is obtained from a set of false cases $drug^{'}_{j}-target^{'}_{j}-disease^{'}_{j}\phantom {\dot {i}\!}$.

#### SemaTyP feature selection

For each path $\pi ^{\ell }_{i}$, a training data (**x**_*i*_,*y*_*i*_) is constructed, where **x**_*i*_ is a vector of semantic types and *y*_*i*_ is a boolean variable indicating whether $\pi ^{\ell }_{i}$ is a positive case. The process of constructing **x**_*i*_ for $\pi ^{\ell }_{i}$ is as follows:

1 $$ \mathbf{x}_{i}=\underset{n=0}{\overset{\ell}{\bowtie}} \Gamma(c_{n})  $$

2 $$ \Gamma(c)= \left\{ \begin{array}{lr} T\_E, & \quad c\in E \\ T\_R, & \quad c\in R \\ \end{array} \right.  $$

**Fig. 3:**
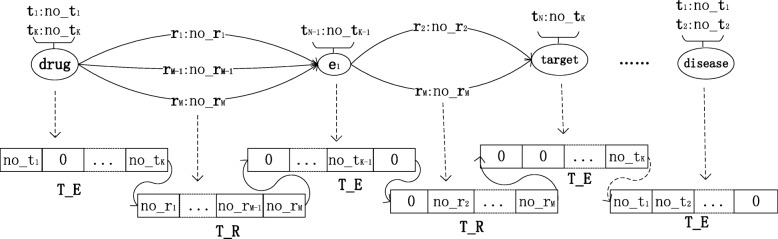
Feature selection of SemaTyP method

The symbol *c* denotes component of path $\pi ^{\ell }_{i}$. *Γ*(*c*) constructs an occurrence number vector of semantic types for *c*. *T*_*E*=[*te*_1_,*te*_2_,…,*te*_*K*_] is a vector of semantic type of entities, the entry of vector is the number of occurrence of corresponding semantic type. Similarly, *T*_*R*=[*tr*_1_,*tr*_2_,…,*tr*_*M*_] denotes a vector of relations and the entry is the number occurrence of corresponding relation. The symbol ⋈ is concatenation of two vectors. For ${\pi }^{\ell }_{i}$, a length of *K*∗(*ℓ*+1)+*M*∗*ℓ* training vector is constructed, where *K* is the length of *T*_*E* and *M* is the length of *T*_*R*. Figure 3 shows an prototype example of constructing one training data. As shown in Fig. 3, the *T*_*E* collects the number of occurrence of all semantic types of corresponding entity, and the *T*_*R* collects the number of occurrence of all relations between its two entities. For the *drug*−*entity*_1_−*target*−*entity*_2_−*disease* case, a length of (*K*∗5+*M*∗4) vector is constructed.

For other path ${\pi }^{m}_{i} (m<\ell)$, it is extended to length *ℓ* by reduplicating entity *target*. For example $\pi ^{m}_{i}=e_{0}r_{0}\mathbf {t}\ldots {r}_{m-1}e_{m}$ is converted to *e*_0_*r*_0_**t***r*_0_**t***r*_0_**t**…*r*_*ℓ*−1_*e*_*ℓ*_, where **t** denotes *target* in this example.

#### Training model

Given a set of training vectors, a logistic regression model is trained to predict conditional probability *P*(*y*|**x**;*θ*). We treat the number of semantic types as features for the logistic regression model.

3 $$ {\theta}_{1}te_{1}+{\ldots}+{\theta}_{K}te_{K}+{\theta}_{K+1}tr_{1}{\ldots}+{\theta}_{K*({\ell}+1)+M*{\ell}}te_{K}  $$

Where the *θ*_*i*_ are appropriate weights for the number of semantic types. The parameter vector ***θ*** is estimated by maximizing a regularized form of the conditional likelihood of y given **x**. In particular, we maximize the objective function

4 $$ O(\boldsymbol{\theta})=\sum_{i}^{2\ell+1}{o_{i}(\theta)-\lambda_{2}|\theta|_{2}}  $$

Where *λ*_2_ controls *L*_2_-regularization to prevent overfitting. *o*_*i*_(*θ*) is the per-instance weighted conditional log-likelihood given by

5 $$ o_{i}(\theta)=y_{i}lnp_{i}+(1-y_{i})ln(1-pi)  $$

Where *p*_*i*_ is the predicted probability

6 $$ p(y_{i}=1|x_{i};\theta)=\frac{exp\left(\Theta^{T}\textbf{x}_{i}\right)}{1+exp\left(\Theta^{T}\textbf{x}_{i}\right)}  $$

The trained logistic regression model is used for discovering candidate drugs for each disease.

#### Implementation of SemaTyP

To evaluate a potential treatment case *drug*_*candidate*_−*target*_*candidate*_−*disease*, first a set of paths *¶*_*candidate*_={*ρ*(*drug*_*candidate*_→*disease*;*target*_*candidate*_,2...*ℓ*)} are obtained by aforementioned method. Then the score of the *drug*_*candidate*_ for disease is:

7 $$ score(drug_{candidate})=\frac{1}{n}\sum_{{\pi}_{i}\in\P_{candidate}}p(y_{i}=1|\chi{(\pi_{i})};\theta)  $$

where *χ*(*π*_*i*_) is the feature selection process for *π*_*i*_ and n is the number of paths in *¶*_*candidate*_.

Since the treatment of the interested disease is unknown, all drugs or chemicals could be one of candidate drugs for the disease. Then all combinations of the drugs and targets are constructed to be hypothetical treatments. Finally, the candidate drugs are ranked by their score.

### Baseline method

Random walk algorithm (RWA) generates finite Markov chains, which can be viewed as random walk on a directed graph [24]. RWA has been employed to resolve a series of problems due to the wide applicability of the algorithm [25]. Here, we compare our method with RWA and other two RWA-based methods, which are considered as the baseline methods.

#### Basic notions of RWA

Let *G*=(*V*,*E*) be a directed graph with *n* nodes and *m* edges. A random walk on *G* is considered as follows: RWA starts at a node *υ*_0_; if *t*-th step is node *υ*_*t*_, RWA moves to the neighbor of *υ*_*t*_ with probability 1/*deg*(*υ*_*t*_). The output of a random walk is a Markov chain (*υ*_*t*_:*t*=0,1,...). We denote by *P*_*t*_ the distribution of *υ*_*t*_:

8 $$ P_{t}(i)=Prob(\upsilon_{t}=i)  $$

We denote by *M*=(*p*_*i*,*j*_)_*i*,*j*∈*Υ*_ the matrix of transition probabilities of this Markov chain. So

9 $$ p_{i,j}= \left\{ \begin{array}{lr} 1/deg(i), & if \quad ij \in E \\ 0, & otherwise \\ \end{array} \right.  $$

Let *A*_*G*_ be the adjacency matrix of *G* and let *D* denote the diagonal matrix with (*D*)_*ii*_=1/*deg*(*i*), then *M*=*DA*_*G*_. The rule of the walk can be expressed by the equation

10 $$ P_{t+1}=M^{T}P_{t}  $$

the distribution of the *t*-th point is viewed as a vector in $\mathbb {R}^{V}$, and hence

11 $$ P_{t}=\left(M^{T}\right)^{t}P_{0}  $$

It follows that the probability $p^{t}_{ij}$ that, starting at *i*, the algorithm reaches *j* in *t* steps is given by the *ij*-entry of matrix *M*^*t*^.

#### Two RWA-based competing methods

In addition to RWA method, we compared our method with two state-of-the-art drug repositioning methods which are NRWRH [26] and TP-NRWRH [27]. NRWRH is a network-based random walk algorithm with restart on heterogeneous network. TP-NRWRH is a two-pass random walk with restart on the drug-disease heterogeneous network. Both of these two methods focus on predicting new targets for a drug of interest.

#### Implementation for drug discovery

**Fig. 4:**
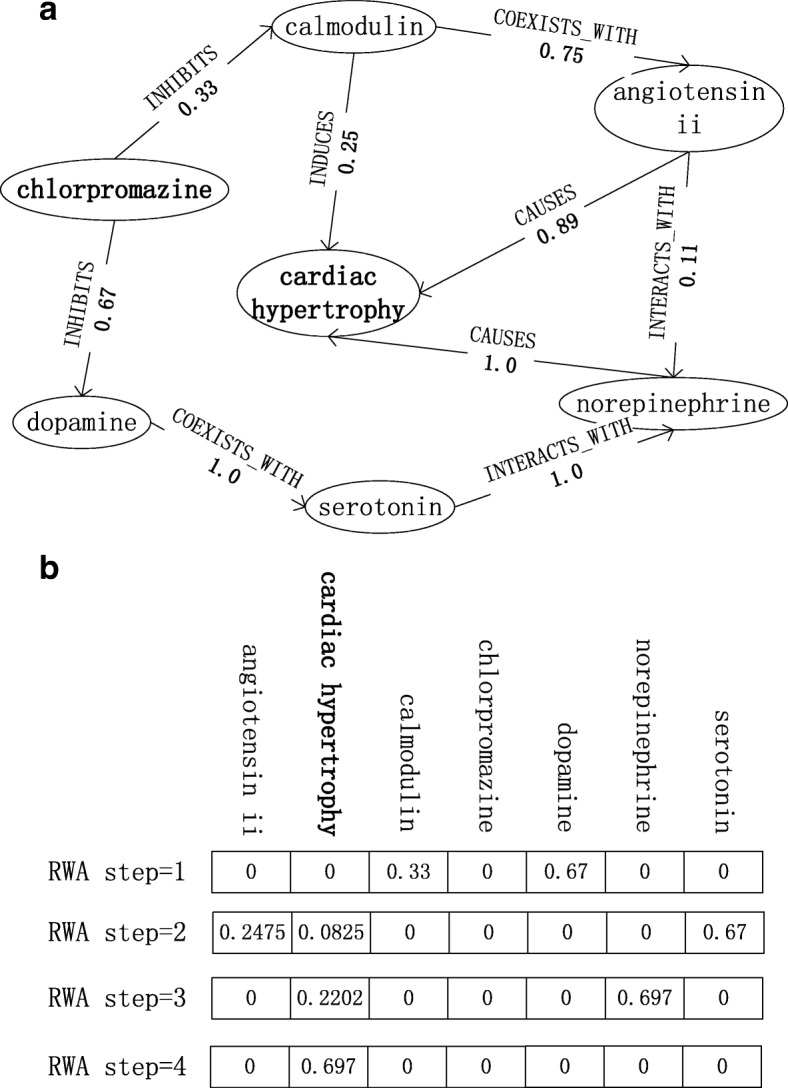
Random Walk Algorithm for drug discovery

To evaluate a potential *drug*_*candidate*_ for treating *disease*_*i*_, the starting node *υ*_0_ of RWA-based methods is set to *drug*_*candidate*_. Figure 4 illustrates an example of evaluating “*chlorpromazine*” to be the treatment of “ *cardiachypertrophy*”. Figure 4a is a weighted semantic graph with 7 nodes and 9 edges. Figure 4b shows the results of RWA with starting node “*chlorpromazine*”. It shows that when the step of RWA is 1, “*chlorpromazine*” can’t reach “ *cardiachypertrophy*”, then the score of “*chlorpromazine*” of step_1 RWA is 0. Similarly, the score of “*chlorpromazine*” for treating “ *cardiachypertrophy*” is 0.697 when the step is 4. For each *disease*_*i*_, RWA scores all candidate drugs of the disease. After that the candidate drugs can be ranked by their scores.

## Results

In this section, we firstly introduce the details of the SemKG and the training data constructed in our experiment. Then, several metrics are introduced to measure the performance of SemaTyP. After that, case studies are conducted to confirm the ability of SemaTyP to find potential drugs for indications.

### The SemKG and training data

#### The SemKG

**Fig. 5:**
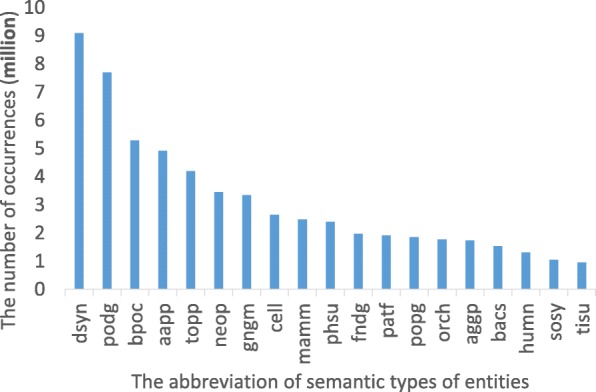
The distribution of semantic types in SemKG

The predications extracted from all abstracts in PubMed (before June 1, 2013) are used to construct the SemKG. Since the performance of SemRep is not perfect: its precision, recall, and F-score are 0.73, 0.55, and 0.63, respectively [28],and the low precision (73%) means many false semantic associations will be returned [12]. We filtered out all the predications that are only extracted once in order to ensure the quality and accuracy of the extracted predications. Table 1 shows the details about the SemKG. Figure 5 is the distribution of top 20 types of entities in the SemKG. For example, the first five types in SemKG are dysn (Disease or Syndrome), podg (Patient or Disabled Group), bpoc (Body Part, Organ, or Organ Component), aapp (Amino Acid, Peptide, or Protein) and topp (Therapeutic or Preventive Procedure).

**Table 1:** The detailed information of SemKG

Materials	Number
PubMed abstracts	22,769,789
Predications	39,133,975
Selected predications	17,651,279
Entities of SemKG	1,067,092
Relations of SemKG	14,419,744
Entity types	133
Relation types	52

#### Training set

In this work, 7144 *drug*−*target*−*disease* are extracted from Therapeutic Target Database (TTD) as true cases (Additional file 1). The *ℓ* is set to 4, *K* is 133 and *M* is 52. Based on the aforementioned construction of training data, 19,230 positive data are obtained. Each data is a length of 873 (133*5 + 52*4) vector. On the other side, for each *drug*−*target*−*disease*, we random replaced the drug, target and disease with other drug, target and disease. If the new triplet doesn’t exist in TTD, then it is considered as a false example, which is denoted as $\phantom {\dot {i}\!}drug^{'}-target^{'}-disease^{'}$. Similarly, 19,230 negative training data is obtained from false cases.

### Evaluation metrics

To systematically evaluate the performance of our method, we conduct ten-fold cross validation and drug rediscovery test.

In the ten-fold cross validation, all training data are randomly divided into ten subsets with equal size. In each cross validation trial, one subset is taken in turn as the test set, while the remaining nine subsets constitute the training set. After performing prediction, each test case is given a predicted score. According to the final predicted scores, the case is assigned a boolean label indicating whether it is a positive case. In this study, the Precision, Recall and F-score are adopted to measure the performance of SemaTyP method.

In our study, drug rediscovery test is performed to evaluate the effectiveness of the SemaTyP when predicting potential drugs for new diseases. For each disease of interest, a list of candidate drugs are constructed to be scored by SemaTyP. Considering the fact that the predicted top-ranked results are more important in practice, we measure the performance of our method in terms of the top-ranked results, i.e. the mean ranking of true therapies and the proportion of correct therapies ranked in the top 10. Usually, it is regarded as more effective if the method can rank more true therapies in top portions.

### Ten-fold cross validation

**Fig. 6:**
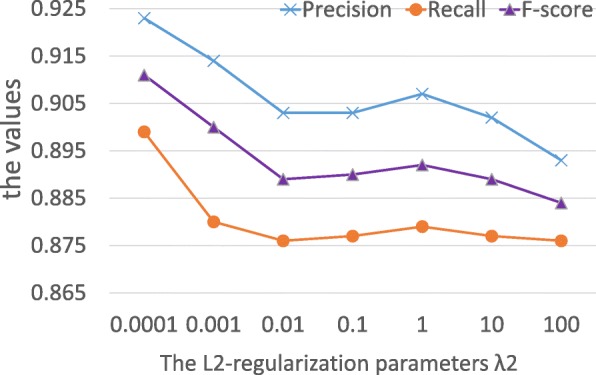
The performance of SemaTyP

We explored a range of values for the *L*_2_-regularization parameters *λ*_2_ using cross validation on the training data. Figure 6 shows that parameter *λ*_2_ ranging from 0.0001 to 100 has little effect on the prediction performance and a small amount of *L*_2_-regularization can slightly improve performance of SemaTyP. In this study, we set the parameter *λ*_2_ to 1.0. The precision, recall and F-score are 0.907, 0.879 and 0.892, respectively. In addition, we also compared the *L*_2_ penalty with Lasso (*L*_1_) regularization [29]. As same to *L*_2_ regularization, the parameter *λ*_1_ of Lasso regularization ranges from 0.0001 to 100. Table 2 shows the comparison results of *L*_1_ and *L*_2_ regularization. The results show that the model achieves higher performance with *L*_2_ regularization. This is because *L*_1_ regularization is often used for feature selection [30] when the number of potentially relevant features is very large. However, in this work the number of features we selected is not large (873).

**Table 2:** The results of logistic regression model with different regularizations

*λ*	Precision		Recall		F-score	
	L1	L2	L1	L2	L1	L2
0.0001	0.908	0.923	0.889	0.899	0.903	0.911
0.001	0.907	0.914	0.878	0.88	0.892	0.900
0.01	0.899	0.903	0.869	0.876	0.884	0.889
0.1	0.905	0.903	0.887	0.877	0.896	0.89
1	0.866	0.907	0.849	0.879	0.857	0.892
10	0.847	0.902	0.837	0.877	0.842	0.889
100	0.823	0.893	0.811	0.876	0.817	0.884

**Fig. 7:**
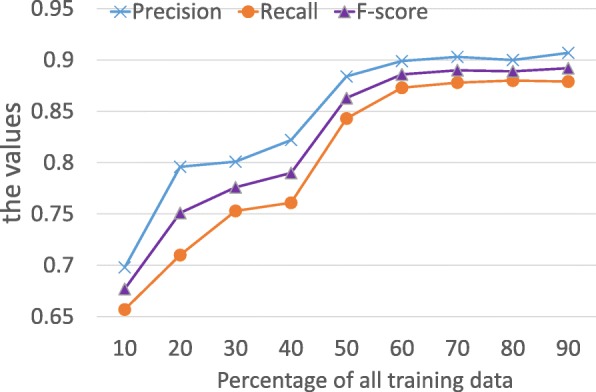
Performance of SemaTyP with different size of training data

We vary the number of training data to see how training data size affects the quality of the model. Figure 7 shows that our method benefits from more training data, and it is especially evident when more than half of all the data are used. Figure 7 shows that the increase in training data significantly improves the performance of SemaTyP when less than 50% training data are used. After that, the increase in training data slightly improves the performance of the method.

Additionally, we vary the settings of *ℓ* to see how pathway length affects the results. The *ℓ* was set to 2, 3 and 4, respectively. Table 3 shows the results of our model with different *ℓ*. It shows that when the *ℓ* is 2, 32 training data was obtained by aforementioned method. It means there are only 32 drugs connect to their indications by directly crossing corresponding targets. We didn’t train the model with the training data, since 32 training data is not enough for training a machine learning model. As shown in Table 3, 1742 data was obtained when *ℓ* is 3. The performance of our model trained by the 1742 data is shown in Table 3. Table 3 shows that the performance of our model with *ℓ* equals 4 is better than *ℓ* equals 3 as expected. As Fig. 7 shows that the increase in training data could significantly improve the performance of our model. When *ℓ* is 3, the size of training data is 9.06% of the training data obtained by *ℓ* equals 4.

**Table 3:** The performance of our model with different training data

*ℓ*	Positive cases	Precision	Recall	F-score
2	32	-	-	-
3	1742	0.791	0.787	0.789
4	19,230	0.907	0.879	0.892

In this work, the *ℓ* is set to a value less than 5, it’s because: 1) Although more training data could be obtained when *ℓ* exceeds 4, Fig. 7 shows that when the training data exceeds certain size, the performance of our method is relatively stable. 2) As *ℓ* increases, longer paths starting from a drug to a disease are obtained. However, more entities in a drug-disease path might reduce the quality of training data. Therefore, in this work, we set the *ℓ* to 4.

### Drug rediscovery test

To evaluate the capability of our method in discovering potential drugs for new diseases, we conduct the drug rediscovery test. In this test, 360 *drug*−*disease* relationships (Additional file 2) are selected from TTD as gold standard to form test set. Each *disease*_*i*_ in test set has one known associated *drug*_*i*_, but the drug mechanism of action is not clear. For each *disease*_*i*_ we randomly selected other 99 drugs or chemicals from TTD as candidate drugs for this disease. We report the mean of those predicted ranks of *drug*_*i*_ and the hits@10, i.e. the proportion of known drugs ranked in the top 10. If the known drug of a disease is not rediscovered, then the score for the drug is set to -1 and the ranking number is 101. Specifically, for *disease*_*i*_ and candidate *drug*_*j*_, 5,785 *drug*_*j*_−*target*_*candidate*_−*disease*_*i*_ are constructed. This is due to that the targets of *disease*_*i*_ are unknown, then each target (protein) in TTD could be the *target*_*candidate*_ of *disease*_*i*_.

For *disease*_*i*_, the comparison methods also scores and ranks all 100 candidate drugs. The step of RWA is set from 1 to 10. The NRWRH and TP-NRWRH methods are configured to their recommended settings in their papers. Table 4 shows the results and the “Not found” column is the number of known drugs which are not found by the method. As we can see from Table 4, there are 262 gold standard drugs are not discovered by RWA_1 (random walk algorithm and the step is set to 1). It means that only 98 (360-262) drugs directly connect to the disease in the SemKG. The “Not found” number decreases when the step number of RWA increases. Table 4 shows that all drugs could be found by RWA when step length exceeds 3. It’s because all drugs could be connected to the disease in the SemKG through a semantic path whose length is greater than 3. Table 4 shows that there are 19 and 17 drugs are not found by NRWRH and TP-NRWRH, respectively. Although the step of the two RWA-based methods is 3, NRWRH and TP-NRWRH are both random walk algorithm with restart. This could result in the diseases fail to reach the appropriate drugs within 3 steps.

**Table 4:** The performance of discovering drugs for disease

Method	Not found	Mean ranking	Hits@10 (%)
RWA_1	262	72.28	28.8
RWA_2	57	26.59	24.46
RWA_3	2	32.45	23.37
RWA_4	0	34.26	19.57
RWA_5	0	35.81	18.75
RWA_6	0	39.14	16.03
RWA_7	0	42.13	14.95
RWA_8	0	44.15	13.59
RWA_9	0	45.69	11.96
RWA_10	0	46.19	11.69
NRWRH	19	31.05	29.72
TP-NRWRH	17	29.87	30.83
Our method	0	**26.31**	**48.61**

Bold values denote the best scores corresponding to specific metric

For the “Mean ranking” column, the worst result is obtained by RWA_1 (72.28), it is due to there are 262 known drugs are not found by RWA_1. As the step length of RWA increases to 2 the meaning ranking decreases to 26.59, it’s because more drugs could be discovered by RWA_2 than RWA_1. But when the step of RWA continues to grow, the mean ranking improves. It’s because although all known drugs could be discovered when the step of RWA exceeds 3, more other candidate drugs also could be found. The more discovered candidate drugs could lead the ranking of true drugs decreasing. Table 4 shows that NRWRH and TP-NRWRH achieve better performance than RWA method, it’s because: 1) The best performance of RWA on “Mean ranking” is achieved when the step is 3, and the step of NRWRH and TP-NRWRH is 3. 2) NRWRH and TP-NRWRH methods integrate biomedical background knowledge to choose next step rather than randomly step to next node.

For “ *Hits@*10”, the value of “ *Hits@*10” decreases when the step of RWA increases. For RWA method, Table 4 shows that RWA_3 and RWA_4 achieve the best performance: 1) almost all drugs could be discovered and 2) the “Mean ranking" value is relatively small and the “ *Hits@*10” is relatively large. In addition, Table 4 shows NRWRH and TP-NRWRH achieve better performance than RWA method. We could see from Table 4, our method achieves the best performance in both tests. The “Mean ranking” of our method is 26.31 and the “ *Hits@*10” is 48.61%. The reasons of our method outperform others are: 1) we could know from Table 4 that when the step of RWA is 3 or 4 the RWA achieves the best performance. Our method could cover all the paths whose length is 2 to 4. 2) Our method scores the semantic path based on the distribution of their semantic types other than only based on the structure of the SemKG.

### Case study

We conduct 12 case studies to demonstrate the efficacy of our methods (Table 5). For each disease, SemaTyP can predict the potential drugs and the corresponding targets simultaneously. For example, TTD has reported that testosterone and ap22408 are known drugs for osteoporosis. These two drugs are ranked 1st and 3rd as potential drugs for osteoporosis by our method. What’s more, SemaTyP also provides corresponding targets for the drugs, which have not been discovered for now. For instance, terikalant is predicated to treat cardiac arrhythmia by acting on actin. Aspirin, is predicted to treat cardiovascular disease by acting lymphoid cell, etc. These prediction instances further confirm that SemaTyP not only has the potential to predict novel drugs for disease, but also could provide potential mechanism of action for the drugs.

**Table 5:** Case study: rediscover known drugs for diseases and provide the new mechanism of action of the drugs

Disease	Target	Drug	Rank
Osteoporosis	col18a1	Testosterone	1
Osteoporosis	Bone metabolism	ap22408	3
Cardiac arrhythmia	Actin	Terikalant	8
Cardiovascular disease	Lymphoid cell	Aspirin	1
Cardiovascular disease	slc5a1	l-nmma	2
Skin allergie	Calprotectin	Mometasone	1
Osteoporosis	Kinase	Calcium-sensing receptor antagonist	3
Anxiety disorder	netrin-1	Benzodiazepine	1
Anxiety disorder	Urotensin ii	Anxiolytic	2
Anxiety disorder	Platelet activating factor	Buspirone	4
Convulsion	epr	Anidulafungin	7
Graft-versus-host disease	fgf21	Flavopiridol	12

## Discussion

To the best of our knowledge, this is the first method that employs knowledge graph for solving LBD tasks. This paper showed that use of implicit semantic types to find drugs from literature can be effective for LBD. Our overall approach however, has several limitations. The first limitation is the construction of knowledge graph - SemKG - relies heavily on effective NLP tools. On one hand, the accuracy of MetaMap reduces in the presence of ambiguity, which leads its inability to resolve word sense disambiguation [20]. On the other hand, although the isolated predications are filtered out in order to improve the quality of the SemKG, there are still considerable number of false predications existing in the knowledge graph, which could lead to our method inferring lower-quality results. In addition, in the process of constructing SemKG, more than half the initial predications are filtered out, which might lead to possible selection biases in the step. The second limitation is SemaTyP relies on the semantic types of nodes and edges to infer associations, hence our method is effective only when the required ontology are easily available. Another limitation is SemaTyP needs to obtain all paths between candidate drug and disease. When the scale of knowledge graph is large, it’s difficult for our method to obtain long paths.

These and other limitations suggest the next steps in this research. In future, high-quality NLP tools need to be developed to improve the quality of SemKG. Additionally, another representation of nodes and edges in SemKG - graph embedding - could be useful for our method to obtain long paths.

## Conclusion

In this work, we have presented a novel method named SemaTyP uncovering the potential associations between drugs (chemicals) and diseases from literature. We first constructed a biomedical knowledge graph by integrating informations extracted from PubMed biomedical literature. Then based on the knowledge graph, we devised a novel model to discover potential drugs and corresponding targets. Finally, we test our method on two different tests. The experimental results show that our method can effectively discover drugs for diseases from literature. Our method has potential to accelerate drug development and benefit the field of target identification.

## Additional files

Additional file 1 Supplementary Data 1. The gold standard *drug*-*target*-*disease* cases used in this work. The 7144 *drug*-*target*-*disease* cases which are extracted from Therapeutic Target Database (TTD) as true cases for constructing training data. (TXT 466 kb)

Additional file 2 Supplementary Data 2. The gold standard *drug*-*disease* cases extracted from TTD. There are 360 *drug*-*disease* relationships are selected from TTD as gold standard to form test data for drug rediscovery test. Each *disease*_*i*_ in test set has one known associated *drug*_*i*_, but the drug mechanism of action is unclear. (TXT 10 kb)

## Abbreviations

CADD: Computer-aided drug discovery/design
HTS: High-throughput screening
LBD: Literature-based discovery
NLP: Natural language processing
TTD: Therapeutic target database

## References

[1] Kore PP, Mutha MM, Antre RV, Oswal RJ, Kshirsagar SS (2012). Computer-aided drug design: an innovative tool for modeling. Open J Med Chem.

[2] Anson BD, Ma J, He J-Q (2009). Identifying cardiotoxic compounds. Genet Eng Biotechnol News.

[3] Zhu T, Cao S, Su P-C, Patel R, Shah D, Chokshi HB, Szukala R, Johnson ME, Hevener KE (2013). Hit identification and optimization in virtual screening: practical recommendations based on a critical literature analysis: miniperspective. J Med Chem.

[4] Morgan S, Grootendorst P, Lexchin J, Cunningham C, Greyson D (2011). The cost of drug development: a systematic review. Health Policy.

[5] Smalheiser NR (2012). Literature-based discovery: Beyond the abcs. J Assoc Inf Sci Technol.

[6] Moffat J. G. (2017). Turning the light on in the phenotypic drug discovery black box. Cell Chem Biol.

[7] Swanson DR (1986). Fish oil, raynaud’s syndrome, and undiscovered public knowledge. Perspect Biol Med.

[8] Sebastian Y, Siew E-G, Orimaye SO (2017). Learning the heterogeneous bibliographic information network for literature-based discovery. Knowl-Based Syst.

[9] Hristovski D, Friedman C, Rindflesch TC, Peterlin B. Exploiting semantic relations for literature-based discovery. In: AMIA Annual Symposium Proceedings. Berlin: American Medical Informatics Association: 2006. p. 349. American Medical Informatics Association. PMC1839258 17238361

[10] Cameron D, Bodenreider O, Yalamanchili H, Danh T, Vallabhaneni S, Thirunarayan K, Sheth AP, Rindflesch TC (2013). A graph-based recovery and decomposition of swanson’s hypothesis using semantic predications. J Biomed Inform.

[11] Cameron DH. A context-driven subgraph model for literature-based discovery (Doctoral dissertation, Wright State University). 2014.

[12] Sang S, Yang Z, Li Z, Lin H (2015). Supervised learning based hypothesis generation from biomedical literature. BioMed Res Int.

[13] Marsi E, Øzturk P, Aamot E, Sizov GV, Ardelan MV. Towards text mining in climate science: Extraction of quantitative variables and their relations. In: Proceedings of the Fourth Workshop on Building and Evaluating Resources for Health and Biomedical Text Processing. Reykjavik: 2014.

[14] Krompa D, Baier S, Tresp V (2015). Type-constrained representation learning in knowledge graphs. International Semantic Web Conference.

[15] Bollacker K, Evans C, Paritosh P, Sturge T, Taylor J (2008). Freebase: a collaboratively created graph database for structuring human knowledge. Proceedings of the 2008 ACM SIGMOD International Conference on Management of Data.

[16] Bizer C, Lehmann J, Kobilarov G, Auer S, Becker C, Cyganiak R, Hellmann S (2009). Dbpedia-a crystallization point for the web of data. Web Semantics: science, services and agents on the world wide web.

[17] Carlson A, Betteridge J, Kisiel B, Settles B, Hruschka Jr ER, Mitchell TM. Toward an architecture for never-ending language learning. In: AAAI Conference on Artificial Intelligence. Georgia: 2010.

[18] Hoffart J, Suchanek FM, Berberich K, Lewis-Kelham E, De Melo G, Weikum G (2011). Yago2: exploring and querying world knowledge in time, space, context, and many languages. Proceedings of the 20th International Conference Companion on World Wide Web.

[19] Korhonen A, Guo Y, Baker S, Yetisgen-Yildiz M, Stenius U, Narita M, Lio P (2014). Improving literature-based discovery with advanced text mining. International Meeting on Computational Intelligence Methods for Bioinformatics and Biostatistics.

[20] Aronson AR, Lang F-M (2010). An overview of metamap: historical perspective and recent advances. J Am Med Inform Assoc.

[21] Aronson AR. Effective mapping of biomedical text to the umls metathesaurus: the metamap program. In: Proceedings of the AMIA Symposium. Washington: 2001. p. 17. American Medical Informatics Association. PMC2243666 11825149

[22] Liu Y, Bill R, Fiszman M, Rindflesch T, Pedersen T, Melton GB, Pakhomov SV. Using semrep to label semantic relations extracted from clinical text. In: AMIA Annual Symposium Proceedings. Chicago: American Medical Informatics Association: 2012. p. 587. American Medical Informatics Association. PMC3540517 23304331

[23] Rindflesch TC, Fiszman M (2003). The interaction of domain knowledge and linguistic structure in natural language processing: interpreting hypernymic propositions in biomedical text. J Biomed Inform.

[24] Lovász L (1993). Random walks on graphs. Comb Paul erdos is eighty.

[25] Weiss G. (1994). Aspects and applications of the random walk (random materials and processes).

[26] Chen X, Liu M-X, Yan G-Y (2012). Drug–target interaction prediction by random walk on the heterogeneous network. Mol BioSyst.

[27] Liu H, Song Y, Guan J, Luo L, Zhuang Z (2016). Inferring new indications for approved drugs via random walk on drug-disease heterogenous networks. BMC Bioinformatics.

[28] Ahler C, Fiszman M, Demner-Fushman D, Lang FM, Thomas CR. Extracting semantic predications from Medline citations for pharmacogenomics. In: Pacific Symposium on Biocomputing. Maui: 2007. p. 209–20. 17990493

[29] Tibshirani R (1996). Regression shrinkage and selection via the lasso. J R Stat Soc Ser B Methodol.

[30] Zou H, Hastie T. J R Stat Soc Ser B Methodol. 2005; 67(2):301–20.

